# Delayed Leaf Senescence by Upregulation of Cytokinin Biosynthesis Specifically in Tomato Roots

**DOI:** 10.3389/fpls.2022.922106

**Published:** 2022-07-06

**Authors:** Noga Glanz-Idan, Michael Lach, Petr Tarkowski, Ondřej Vrobel, Shmuel Wolf

**Affiliations:** ^1^The Robert H. Smith Institute of Plant Sciences and Genetics in Agriculture, The Robert H. Smith Faculty of Agriculture, Food and Environment, The Hebrew University of Jerusalem, Rehovot, Israel; ^2^Center of the Region Haná for Biotechnological and Agricultural Research, Czech Advanced Technology and Research Institute, Palacký University, Olomouc, Czechia; ^3^Center of the Region Haná for Biotechnological and Agricultural Research, Department of Genetic Resources for Vegetables, Medicinal and Special Plants, Crop Research Institute, Olomouc, Czechia

**Keywords:** cytokinin, isopentenyltransferase, photosynthesis, shoot-to-root ratio, *Solanum lycopersicum*

## Abstract

Cytokinins (CKs) regulate numerous plant developmental processes, including photosynthesis and leaf senescence. Isopentenyltransferase (IPT) is a rate-limiting enzyme in the CK-biosynthesis pathway. We overexpressed ipt under tissue-specific promoters to study the long-range effect of CK on the functioning of tomato source leaves. Photosynthetic activity over time provided the measure for leaf aging. Significantly delayed leaf senescence was observed in plants expressing ipt under a root-specific promoter, but not in those expressing the gene under a source leaf-specific promoter. The root-derived influence on leaf aging was further confirmed by grafting experiments. CK concentration in source leaves of both transgenic lines increased significantly, with different proportions of its various derivatives. On the other hand, root CK concentration was only slightly elevated. Nevertheless, the significant change in the proportion of CK derivatives in the root indicated that CK biosynthesis and metabolism were altered. Partial leaf defoliation upregulates photosynthetic rate in the remaining leaf; however, overexpression of ipt in either tissues eliminated this response. Interestingly, stem girdling also eliminated the photosynthetic response. Taken together, our findings suggest that leaf senescence is regulated by a CK-mediated root–shoot communication network. We propose that CK-mediated signal is translocated to the leaf via the xylem where it alters CK biosynthesis, resulting in delayed senescence.

## Introduction

Leaf senescence is an active and organized process described by a decrease in photosynthetic rate, protein degradation, a drop in chlorophyll content, and nutrient remobilization from source tissues, such as mature leaves, to sink tissues, such as roots, fruit, or young leaves (Smart, 1994; Gan and Amasino, 1997). This complex process seems to be directly related to a decrease in cytokinin (CK) concentration and activity (Gan and Amasino, 1996).

CKs are small, adenine-derived molecules that are formed primarily in the roots, and move acropetally to promote shoot development and stress tolerance, and to delay senescence (Müller and Sheen, 2007; Domagalska and Leyser, 2011). CKs preserve sink activity of old leaves by inducing changes in nitrogen mobilization and enhancing protein concentration *via* decreased protein degradation (Jordi et al., 2000; Criado et al., 2009). The various forms of CKs can be classified into four groups, depending on their side-chain structure: isopentenyladenine (iP) type, *trans*-zeatin (*t*Z) type, *cis*-zeatin (*c*Z) type, and aromatic CKs. Both iP-type and *t*Z-type CKs are biologically important (Matsumoto-Kitano et al., 2008). CK biosynthesis starts with the attachment of dimethylallyl diphosphate (DMAPP) to ATP catalyzed by the enzyme isopentenyltransferase (IPT), resulting in N^6^-isopentenyladenine ribotides (Sakakibara, 2006). Next, hydroxylation of the isoprenoid side chain by cytochrome P450 converts the iP form to the *t*Z form (Takei et al., 2004). Both iP and *t*Z ribonucleosides are considered long-distance signals because of their mobility through the vasculature. The iP type is more abundant in the phloem sap, signifying its transport from source to sink organs, whereas the *t*Z type is predominant in the xylem sap, suggesting that it is trafficked mainly from roots to shoots (Hirose et al., 2008; Matsumoto-Kitano et al., 2008). Both mobile ribonucleosides can be converted to free bases at the site of their functioning, in a single enzymatic step catalyzed by the LONELY GUY (LOG) family of cytokinin nucleoside 5′ monophosphate phosphoribohydrolases (Kuroha et al., 2009). Negative feedback on CK levels is activated by either conjugation to glucose (Bajguz and Piotrowska, 2009) or irreversible degradation by cytokinin oxidases/dehydrogenase (CKXs; Schmülling et al., 2003; Werner et al., 2006).

We recently reported that reducing the source-to-sink ratio by partial defoliation or leaf shading results in upregulation of photosynthesis and delayed senescence of the remaining leaf (Glanz-Idan et al., 2020). Additional observed phenotypic changes, such as thickening and greening of the remaining leaf, suggested involvement of CKs in the response to partial defoliation. We proposed that root-derived CK acts as a long-distance signal influencing leaf activity. Indeed, these observed partial defoliation effects were associated with significant alterations in CKs metabolism in the roots.

In the current study, we provide experimental evidence for the specific effect of CK biosynthesis in the roots on source leaf functioning. Overexpression of *IPT* under the control of a root-specific promoter significantly affected shoot phenotype and delayed leaf senescence, whereas overexpression of this gene predominantly in source leaves had only a marginal and nonsignificant effect. Grafting experiments further demonstrated that upregulation of photosynthesis after partial defoliation can be mimicked by overexpression of IPT solely in the rootstock. In addition, girdling experiments indicated that proper shoot–root phloem communication is required for the effect of partial defoliation on the activity of source leaves. Collectively, our findings suggest that alteration in CK metabolism in the roots may provide a valuable means to delay leaf senescence by modifying long-distance root–shoot signaling.

## Materials and Methods

### Plant Material

Tomato (*S. lycopersicum* L. cv. M82) served as the control and background for production of the various transgenic plant lines. All transgenic lines were created using the PoP/LhG transactivation system (Moore et al., 1998). The activator lines expressed the *Arabidopsis* MDK4-20 (AtMDK4) root cap-specific promoter (Lilley et al., 2011) or the *Escherichia coli* cytosolic fructose-1,6-bisphosphatase (cyFBPase) promoter, which directs expression mainly in the mesophyll cells of source leaves (Lloyd et al., 1991). The genes were cloned upstream of the synthetic transcription factor LhG4, and transgenic tomato plants were produced using conventional *Agrobacterium*-mediated transformation (McCormick, 1991). The operator lines expressing either *AtIPT* or *AtCKX3* under an operator promoter (OP) from *E. coli* were kindly provided by Prof. Naomi Ori, The R.H. Smith Institute of Plant Sciences, Rehovot (Shani et al., 2010). Four different transgenic lines: MDK:CKX, MDK:IPT, FBPase:CKX, and FBPase:IPT resulted from crosses between the activator lines AtMDK:LhG4 and cyFBPase:LhG4 with the operator lines OP:AtIPT7 and OP:AtCKX3.

### Growth Conditions and Partial Defoliation

Plants were sown in trays filled with 70% coconut fiber, 30% vent materials, fertilizers, and nutrients in a temperature-controlled growth chamber. Air temperatures were 25/18 ± 2°C day/night, respectively, and light intensity was 180 μmol m^−2^ s^−1^. Photoperiod was set to 12 h. Seedlings (4 weeks old) were transferred to 1-L pots and grown in a greenhouse at 28/20 ± 2°C (day/night, respectively) under natural light (1,000–1,200 μmol m^−2^ s^−1^). After additional 3–4 weeks, partial defoliation was performed at the reproductive stage, 1 week after anthesis of the first flower. All leaves, except for the measured one, were carefully cut near the stem with a sharp surgical knife.

Measurements of photosynthesis were performed in plants at the reproductive stage. Development of MDK:IPT plants was slower, and for comparison with control plants they were sown 3 weeks earlier such that all measurements were performed with plants at a similar physiological stage.

### Grafting and Girdling

Grafting was performed with 3-week-old seedlings by diagonal stem cut 3 cm from the shoot apical meristem. The scions were placed above the rootstocks and secured using a grafting clip. New grafts were covered with clear plastic for 5 days. Clips were removed 3 weeks later when the graft union closed and plants were transferred to a temperature-controlled greenhouse.

Girdling was performed by a 2-mm cut around the stem between the first and second joint. On 2 days later, a second cut was made about 3 mm above the first one to prevent recovery of the stem tissues. Girdling effectiveness was verified by petiole infusion of two leaves, numbers 3 and 4 above the girdling point. The leaves were cut 3–4 cm from the main stem, and the petioles were fed with 2% blue dye according to Lin et al. (2011). On 3 days after the second girdling, several stem sections were taken above and below the girdling point to monitor dye movement *via* the phloem. Images were taken using a light binocular (Supplementary Figure S1).

### Gas-Exchange Measurements and Chlorophyll Determination

Gas exchange was measured with a LI-6800 portable gas-exchange system (LI-COR Biosciences). Photosynthesis, CO_2_ concentration in the substomatal cavities (Ci), stomatal conductance, and transpiration rate were measured in the greenhouse. Measurements were performed at around noon under a constant CO_2_ concentration of 400 ppm, chamber relative humidity of 55%, and constant photosynthetic photon flux density (PPFD) of 1,200 μmol m^−2^ s^−1^.

Chlorophyll content was determined as described previously (Moran, 1982). Five leaf disks (50.24 mm^2^ each) were excised between the minor ribs, weighed for specific weight calculation, and placed in 10 ml N,N-dimethylformamide solution for 48 h at 4°C in the dark. The absorbance of the supernatant was measured at 647 and 664 nm, and chlorophyll content was computed using the formula: chlorophyll a + b (mg ml^−1^) = 10 × (0.02027 × A_647_ + 0.00704 × A_664_).

### CKs Extraction and Analyses

For CK analyses, plant material (150 mg) was ground by vibration mill and extracted with Bieleski solvent (Bieleski, 1964). Stable isotope-labeled CKs internal standards (OlChemIm) were added before extraction and CKs were purified using SCX SPE columns (Agilent) and quantified by liquid-chromatography/positive-ion electrospray tandem mass spectrometry in multiple reaction monitoring mode (Holubová et al., 2018).

### Statistical Analysis

ANOVA and mean comparison were performed by Student’s *t*-test to compare two variables or by Tukey–Kramer HSD test for multivariate analysis (*α* < 0.05), using JMP Pro version 14 (SAS Institute).

## Results

### Manipulation of CKs Biosynthesis in Specific Plant Tissues

Cytokinin biosynthesis was manipulated in transgenic tomato plants by overexpressing either *AtIPT7* or *AtCKX3* genes. The root-cap-specific promoter *AtMDK4* was used (Lilley et al., 2011; Supplementary Figure S2), and the *FBPase* promoter was aimed at directing gene expression mainly to leaf mesophyll cells (Lloyd et al., 1991). Overexpression of *IPT* primarily in source leaves (FBPase:IPT) resulted in smaller plants with a *ca.* 30% decrease in shoot and root weight (Table 1). However, expression of this gene specifically in the roots (MDK:IPT) had a much more severe effect on plant development and growth (Table 1; Supplementary Figure S3 A). These plants were stunted, reaching a total weight of about 20% of the control plants. It is interesting to note that when expressed in the roots, the effect of *IPT* on shoot weight was much more pronounced than its effect on root weight, resulting in a significantly higher root-to-shoot ratio (Table 1). Rate of leaf senescence was determined by the reduction in photosynthetic activity over time. As expected, a significant decrease of over 50% in photosynthetic rate was measured in 30-day-old control M82 source leaves (Table 2). The decline in 30-day-old leaves of the three MDK:IPT leaves was in the range of 10%–30%, indicating significantly slower senescence rate (Table 2). It is important to note that a similar decrease in photosynthetic rate with leaf aging was measured in M82 plants and in those expressing *IPT* under *FBPase*, a source leaf-specific promoter (Supplementary Figure S4). These results established that overexpression of *IPT* specifically in the roots significantly inhibits leaf senescence.

**Table 1:** Dry weight accumulation and leaf area of transgenic tomato plants expressing *AtIPT* under source leaf-specific promoter *FBPase* or root cap-specific promoter *AtMDK*.

	Shoot dry weight (g)	Root dry weight (g)	Root:shoot ratio	Average leaf size (cm^2^)
M82	21.6 ± 1.5 a	15.5 ± 0.7 a	0.73 ± 0.06	117 ± 16
FBPase:IPT	13.4 ± 2.4 b	10.2 ± 1.3 b	0.79 ± 0.05	93 ± 11
M82	19.3 ± 2.4 a	15.8 ± 1.4 a	0.86 ± 0.1 a	125 ± 19 a
AtMDK:IPT	1.8 ± 0.4 b	4.5 ± 0.8 b	3.04 ± 0.4 b	24 ± 15 b

Data represent means (±SE) of six replicates of two independent experiments comparing each transgenic line with the control variety M82.

Different lowercase letters indicate significant differences between the control and the respective transgenic plants by Student’s *t*-test, *p* < 0.05.

**Table 2:** Decline in photosynthetic rate with aging of source leaves of the control variety M82 and three independent transgenic tomato lines expressing Isopentenyltransferase (*IPT*) under the *AtMDK* (MDK:IPT) promoter.

Plant line	Photosynthetic rate (μmol CO_2_ m^−2^ s^−1^)		(% of initial rate)
	Day 0	Day 30	
M82	16.8 ± 1.1	7.6 ± 0.3	46 ± 5
MDK:IPT3	12.1 ± 1.3	8.4 ± 1.4	70 ± 11*
MDK:IPT4	12.3 ± 0.9	8.2 ± 0.7	67 ± 6*
MDK:IPT5	15.6 ± 1.2	13.7 ± 0.8	90 ± 9**

Values represent the mean measured values of six replicates at day 0 (fully expanded leaf), 30-day-old leaves and the percentage of the values measured in old leaves from those measured in young fully expanded leaves. Plants were grown in a temperature-controlled greenhouse (25/18°C day/night). Data represent means (±SE).

Student’s *t*-test was used to compare mean values of four plants. Asterisks indicate significant differences between each transgenic line and the control variety M82 by Student’s *t*-test at *p* < 0.05 (*) and *p* < 0.01 (**).

**Figure 1:**
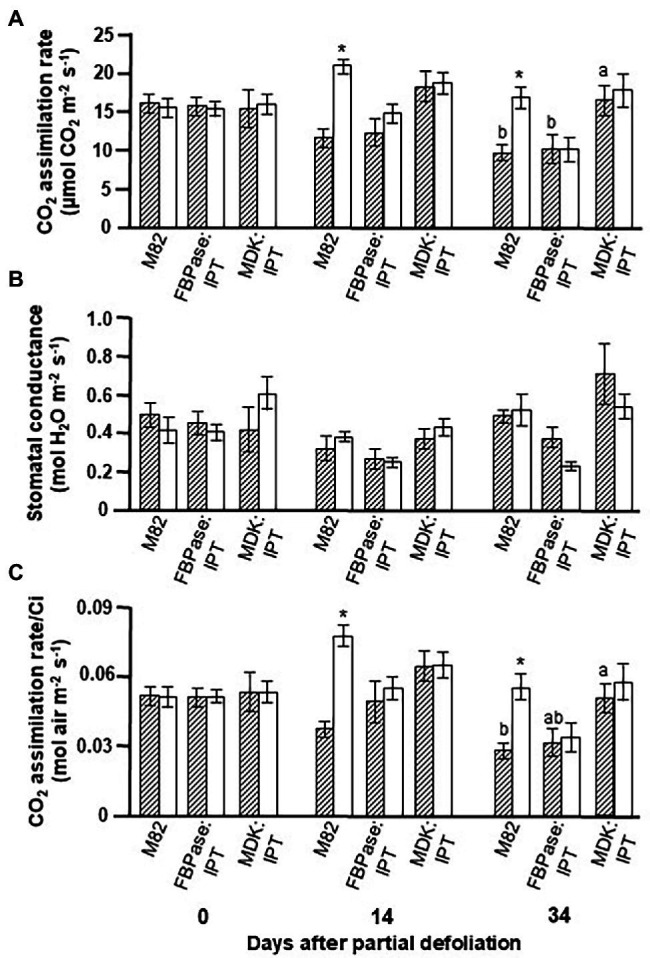
Effect of enhanced cytokinin biosynthesis on the photosynthetic activity response to partial defoliation. Photosynthetic rate **(A)**, stomatal conductance **(B)**, and rate of photosynthesis per internal CO_2_ concentration (Ci; **C**) of source leaves in nondefoliated plants (hatched bars) and remaining tomato leaves after partial defoliation of the other leaves (empty bars). Defoliation was performed on mature plants at the reproductive stage of variety M82 and transgenic tomato plants expressing *IPT* under *FBPase* (FBPase:IPT, plant line #2) or *AtMDK* (MDK:IPT, plant line #5) promoters. Plants were grown in a temperature-controlled greenhouse (25°C/18°C day/night). Data represent means (±SE) of six biological replicates. Different letters indicate significant differences between the tomato lines on a specific date (*p* < 0.05) by Tukey HSD test. Asterisks indicate significant differences between partially defoliated and nondefoliated same tomato line at *p* < 0.05 by Student’s *t*-test.

Plant lines MDK:IPT #5 and FBPase:IPT #2 were selected for further experiments, aimed at exploring the response of photosynthetic activity to partial defoliation. Here again, the photosynthetic rate in MDK:IPT leaves remained constant for about 5 weeks (Figure 1A). The higher photosynthetic activity of plant line MDK:IPT after 34 days was associated with higher carboxylation efficiency, which was reflected by the rate of photosynthesis per internal CO_2_ concentration (Ci), and not due to significant differences in stomatal conductance (Figures 1B,C).

We previously reported that partial leaf defoliation upregulates photosynthetic rate in the remaining leaf, and that this upregulation is due to a higher rate of photosynthesis per Ci (Glanz-Idan et al., 2020). A similar phenomenon was observed in the current study (Figure 1). Interestingly, overexpression of *IPT*, in source leaves or roots, eliminated the response to partial defoliation. A similar reduction in photosynthesis with leaf aging was measured in FBPase:IPT plants, whereas similar delayed senescence was measured in MDK:IPT leaves in control and partially defoliated plants.

**Figure 2:**
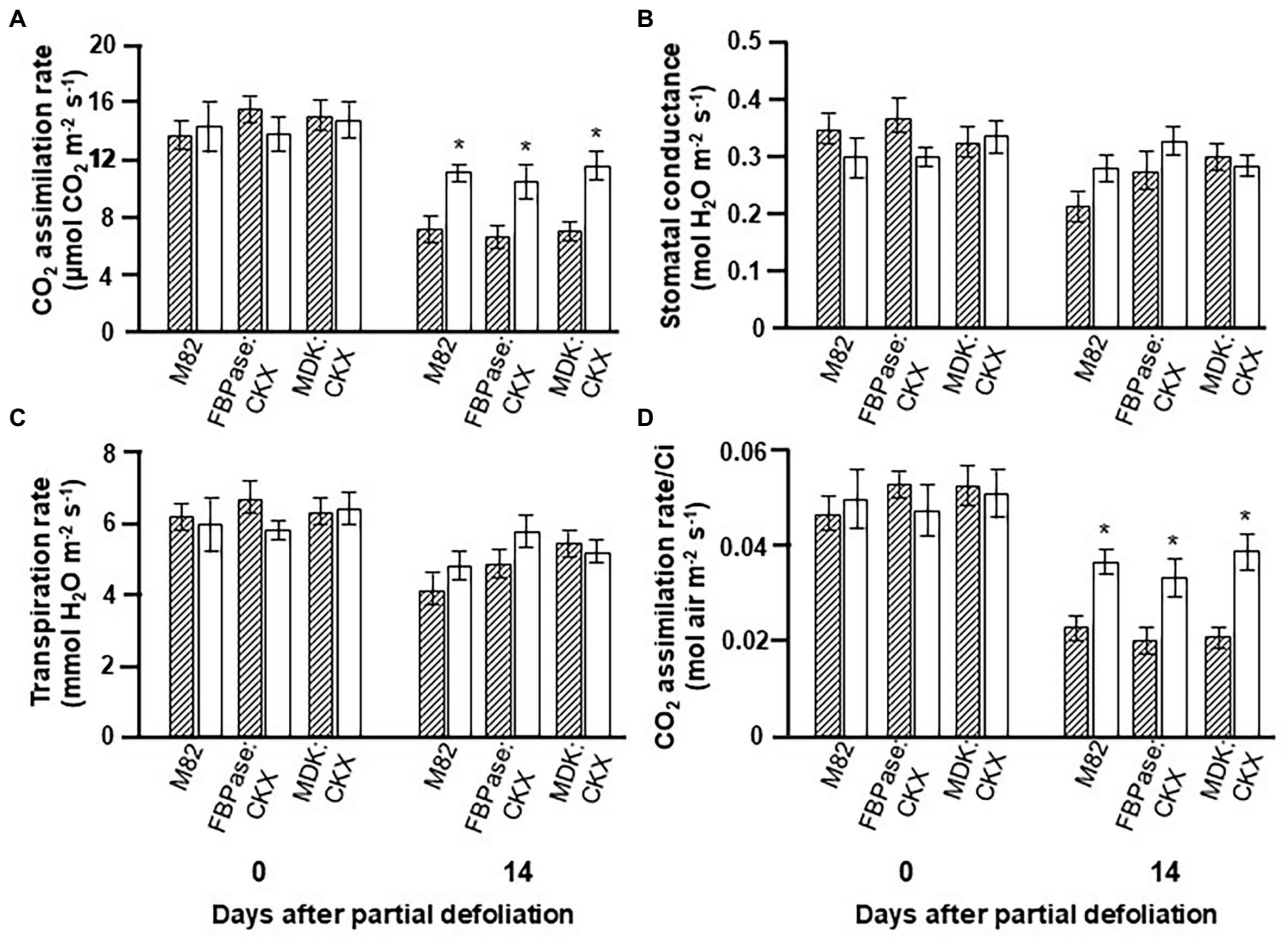
Effect of reduced cytokinin biosynthesis on the photosynthetic activity response to partial defoliation. Photosynthetic rate **(A)**, stomatal conductance **(B)**, transpiration rate **(C)**, and rate of photosynthesis per internal CO_2_ concentration (Ci; **D**) of source leaves in nondefoliated plants (hatched bars) and the remaining tomato leaves after partial defoliation of the other leaves (empty bars). Defoliation was performed on mature plants at the reproductive stage of variety M82 and transgenic tomato plants expressing *CKX* under *FBPase* (FBPase:CKX) or *AtMDK* (MDK:CKX) promoters. Plants were grown in a temperature-controlled greenhouse (25°C/18°C day/night). Student’s *t*-test was used to compare mean values of six plants. Asterisks indicate significant differences between partially defoliated and nondefoliated same tomato line at *p* < 0.05.

To reduce CK concentration in either the source leaves or the roots, an additional set of transgenic plants was created that overexpressed *CKX* under the indicated tissue-specific promoters. The phenotype of these plants was relatively similar to the control variety, with MDK:CKX being somewhat taller (Supplementary Figure S3 B). The photosynthetic rate in plants expressing *CKX* under either promoter was similar to that measured for the control plants (Figure 2). The other parameters, such as stomatal conductance, transpiration rate, and photosynthesis per Ci, were also similar for the transgenic and control plants, even when the leaves were old (after 14 days). Here again, partial defoliation caused upregulation of photosynthesis in control and *CKX-*overexpressing lines under both promoters (Figure 2).

### CKs Concentration in Roots and Leaves of *IPT*- and *CKX*-Overexpressing Plants

**Figure 3:**
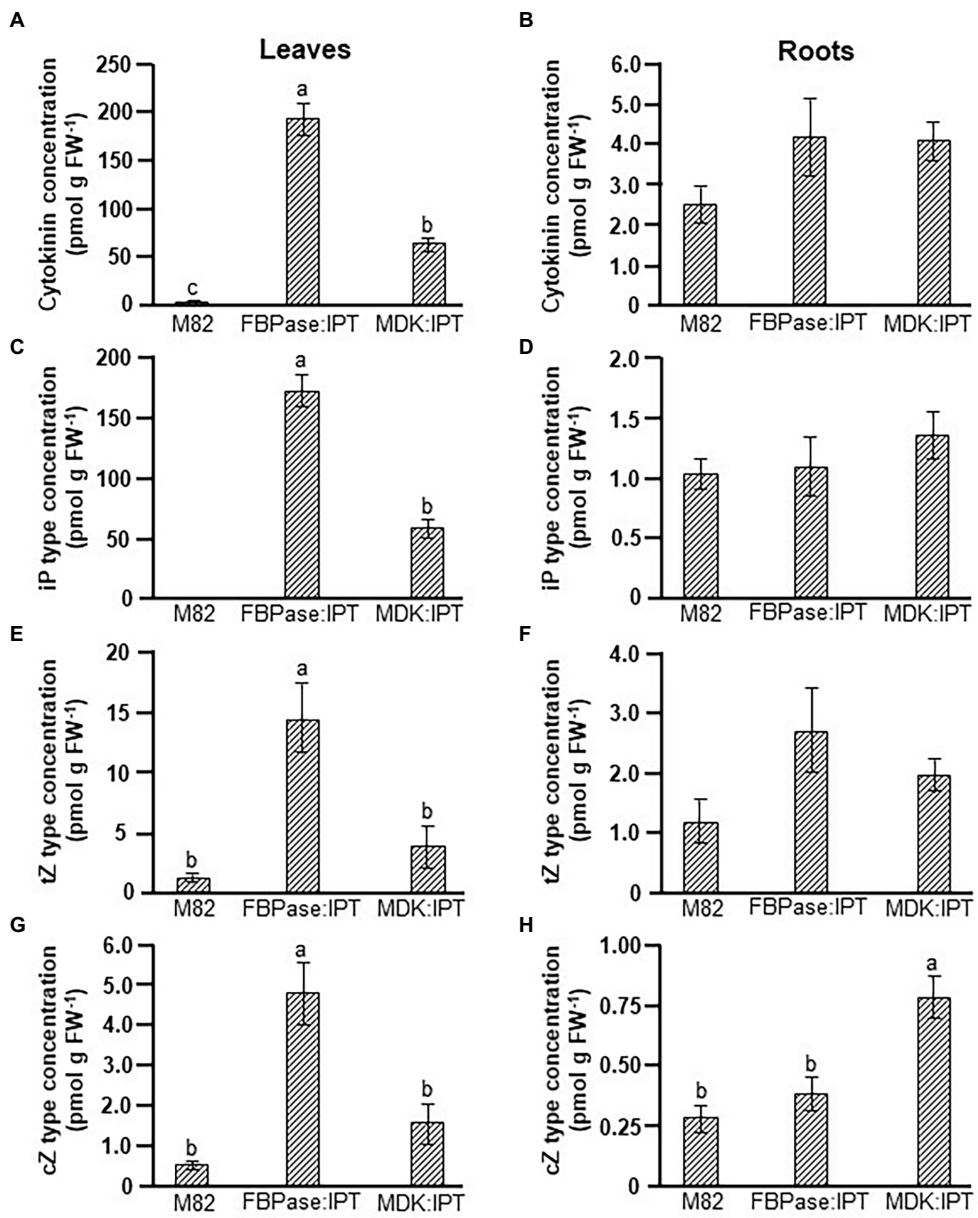
Elevation of cytokinin concentration in tomato plants expressing *IPT* specifically in roots and source leaves. Total cytokinin **(A,B)**, iP type **(C,D)**, *t*Z type **(E,F),** and *c*Z type **(G,H)** concentrations in source leaves **(A,C,E,G)**, and roots **(B,D,F,H)** of control variety M82 and transgenic tomato plants expressing *IPT* under *FBPase* or *AtMDK* promoters (FBPase:IPT, plant line #2 and MDK:IPT, plant line #5, respectively). Plants were grown in a temperature-controlled greenhouse (25°C/18°C day/night). Data represent means (±SE) of six biological replicates. Different letters indicate significant differences between plant lines at *p* < 0.05 by Tukey HSD test.

A major influence on source leaf functioning was observed when *IPT* was expressed under the root-specific promoter, whereas no influence was observed when *CKX* was expressed under either tissue-specific promoters. Analyses of CKs levels confirmed significant differences in total CKs concentrations between *IPT*-overexpressing and control plants (Figures 3A,B). As expected, the total concentration of CKs in leaves of FBPase:IPT plants was about 100-fold higher than that in control plants, mainly due to accumulation of iP-type CKs (Figures 3A,C). Concentrations of *t*Z- and *c*Z-type CKs were also significantly higher in FBPase:IPT plants, about 10-fold higher than that measured in control plants (Figures 3E,G). Total CKs in leaves of MDK:IPT plants were about 25-fold higher than those in control leaves, also attributed to accumulation of iP-type CKs, although not to the same extent as FBPase:IPT leaves (Figure 3C). Total CKs concentration in the roots was upregulated in both FBPase:IPT and MDK:IPT plants (Figure 3B). Interestingly, concentrations of total CKs, and of iP-type and *t*Z-type CKs, were similar in the roots of plants overexpressing *IPT* under the root- and shoot-specific promoters (Figures 3B,D,F). However, the concentration of *c*Z-type CKs in the roots was significantly higher in MDK:IPT plants than in both control and FBPase:IPT plants (Figure 3H).

More detailed analyses of the various CKs forms revealed that most of the CKs accumulated in the leaves of FBPase:IPT plants were in the form of iP-riboside (iPR). This form was also dominant in leaves of MDK:IPT plants (Supplementary Figure S5). Overexpression of *IPT* in leaves did not have a significant effect on the concentration of specific CK forms in the roots (Supplementary Figure S6). However, a significant increase in the root concentrations of iP, iP-9-glucoside (iP9G), *t*Z-9-glucoside (*t*Z9G), and *c*Z and *c*Z-riboside (*c*ZR) was evident in MDK:IPT plants. It is important to note that when comparing root CKs concentrations between the two transgenic lines, significantly higher concentrations of iP9G, *t*Z9G, and *c*ZR were measured in MDK:IPT plants (Supplementary Figure S6).

Overexpression of *AtCKX* under either source leaf or root specific promoters did not have a significant influence on CKs concentration (Supplementary Tables S1, S2). It is important to note that the expression of *AtCKX* in the different transgenic tomato lines was validated and was found to be tissue-specific in the two transgenic tomato lines: FBPase:CKX and MDK:CKX (Supplementary Figure S7). Some minor changes were observed in the proportion of the various CKs forms, in both leaves and roots, but total CKs concentrations were similar in the roots and source leaves of all plant lines.

### Effect of Root-Derived CKs on the Functioning of Source Leaves in Heterografted Plants

**Figure 4:**
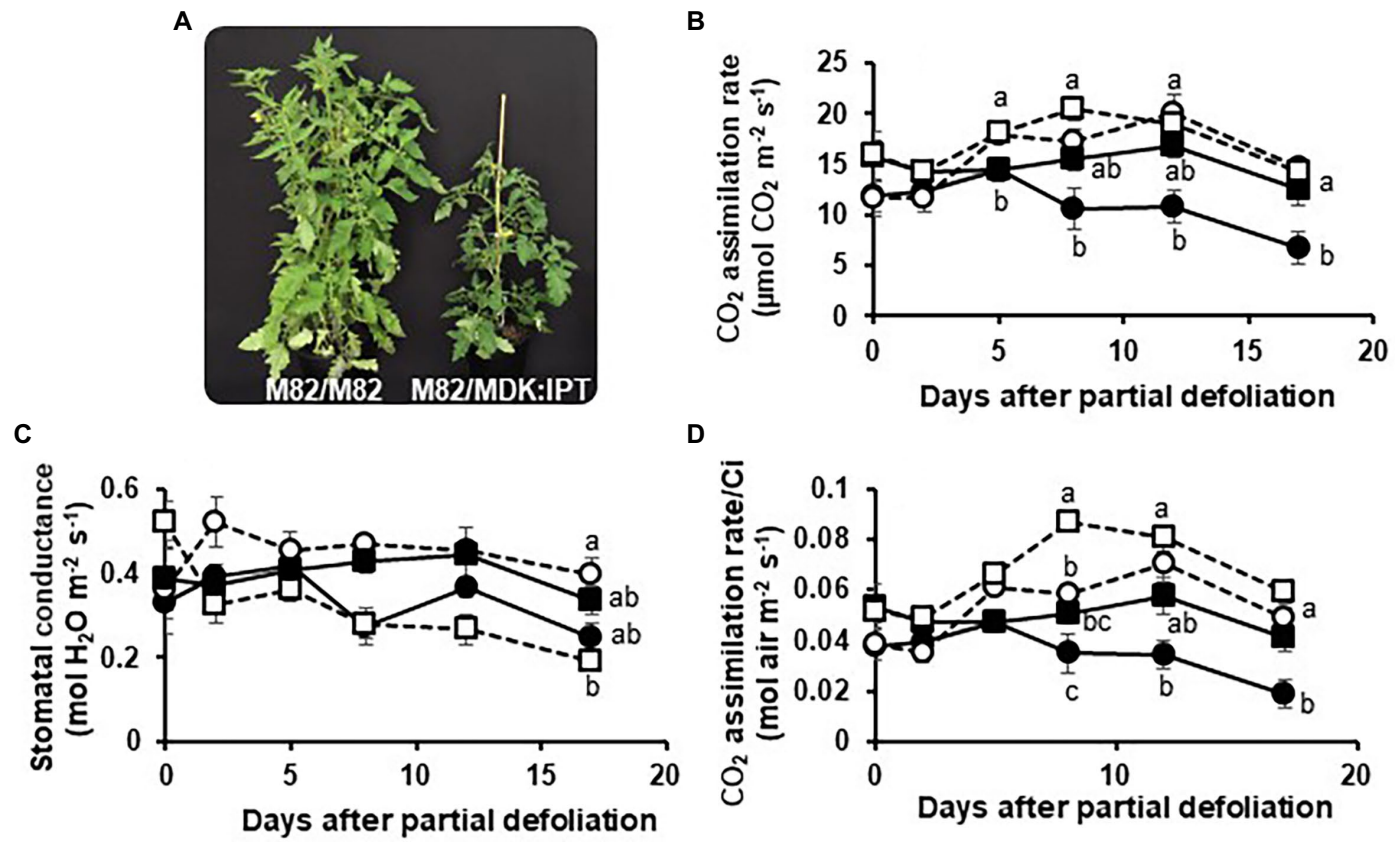
Enhanced cytokinin biosynthesis in the roots delays leaf senescence in grafted plants. Picture of the homografted M82/M82 (left) and heterografted M82 scions over MDK:IPT rootstocks (right; **A**), photosynthetic rate **(B)**, stomatal conductance **(C)**, and the rate of photosynthesis per internal CO_2_ concentration (Ci; **D**) of remaining tomato leaves after partial defoliation of the other leaves (empty symbols). Defoliation was performed on either homografted (circles) or heterografted (squares) plants grown in a temperature-controlled greenhouse (25°C/18°C day/night). Dashed lines and empty symbols represent partially defoliated plants; solid lines and full symbols represent nondefoliated plants. Student’s *t*-test was used to compare mean values of six plants. Different letters indicate significant differences at *p* < 0.05.

*Solanum* plants, such as tomatoes, are known for their ability to form adventitious roots from root primordia in the stem (Vidoz et al., 2010). One might argue that the influence of *IPT* on leaf functioning, when overexpressed under a root-specific promoter, is due to its expression in the stem root primordia. To examine this possibility, a set of heterografted plants were produced. M82 scions were grafted on MDK:IPT rootstocks, below the cotyledons. Homografted M82 plants served as controls (Figure 4A). As expected, a gradual decrease in photosynthetic rate was associated with leaf aging in the homografted control plants, resulting in an over 40% reduction within 17 days (Figure 4B). Similar to the previous results (Figure 1), partial defoliation upregulated photosynthesis in these plants and delayed leaf senescence. However, when grafted on MDK:IPT rootstocks, photosynthetic rate of the control M82 variety source leaves remained constant during this time period. Interestingly, in these heterografted plants, no response to partial defoliation was observed (Figure 4B). More detailed analyses of photosynthetic activity indicated that elevation of photosynthesis in the heterografted plants was not associated with differences in stomatal conductance (Figure 4C) but with photosynthetic rate per Ci (Figure 4D). Source leaf morphology was not affected by the expression of *IPT* in the rootstocks, as indicated by similar specific leaf weight (Table 3). Nevertheless, these rootstocks eliminated the reduction of chlorophyll concentration in 18-day-old leaves (Table 3).

**Table 3:** Specific leaf weight and chlorophyll concentration in old source leaves of M82 scions grafted on control M82 or transgenic MDK:IPT rootstocks.

	M82/M82	M82/MDK:IPT
Specific leaf weight of 18-day-old leaves (mg cm^−2^)	21.7 ± 0.7	23.6 ± 2.5
Chlorophyll concentration in fully expanded leaves (μg cm^−2^)	40.2 ± 3.1	40.4 ± 2.6
Chlorophyll concentration in 18-day-old leaves (μg cm^−2^)	33.3 ± 4.0 b	45.8 ± 4.1 a

Samples were taken from fully expanded source leaves and 18 days after full expansion. Data represent means (±SE) of six replicates.

Different letters indicate significant differences between homografted and heterografted plants by Student’s *t*-test, *p* < 0.05.

Senescence rate was also measured in a reciprocal grafting, where MDK:IPT scions were grafted on M82 control plants. A *ca.* 40% reduction in photosynthetic rate within 17 days was observed in MDK:IPT scions. A senescence rate similar to that of the homografted M82 plants was observed during the first 2 weeks, with slightly lower rate during week 3 (Supplementary Figure S8).

Taken together, these results established that development and functioning of source leaves are more affected by enhanced CKs biosynthesis in the roots than in those leaves.

### Upregulation of Photosynthesis Is Controlled by Long-Distance Shoot–Root Signaling

**Figure 5:**
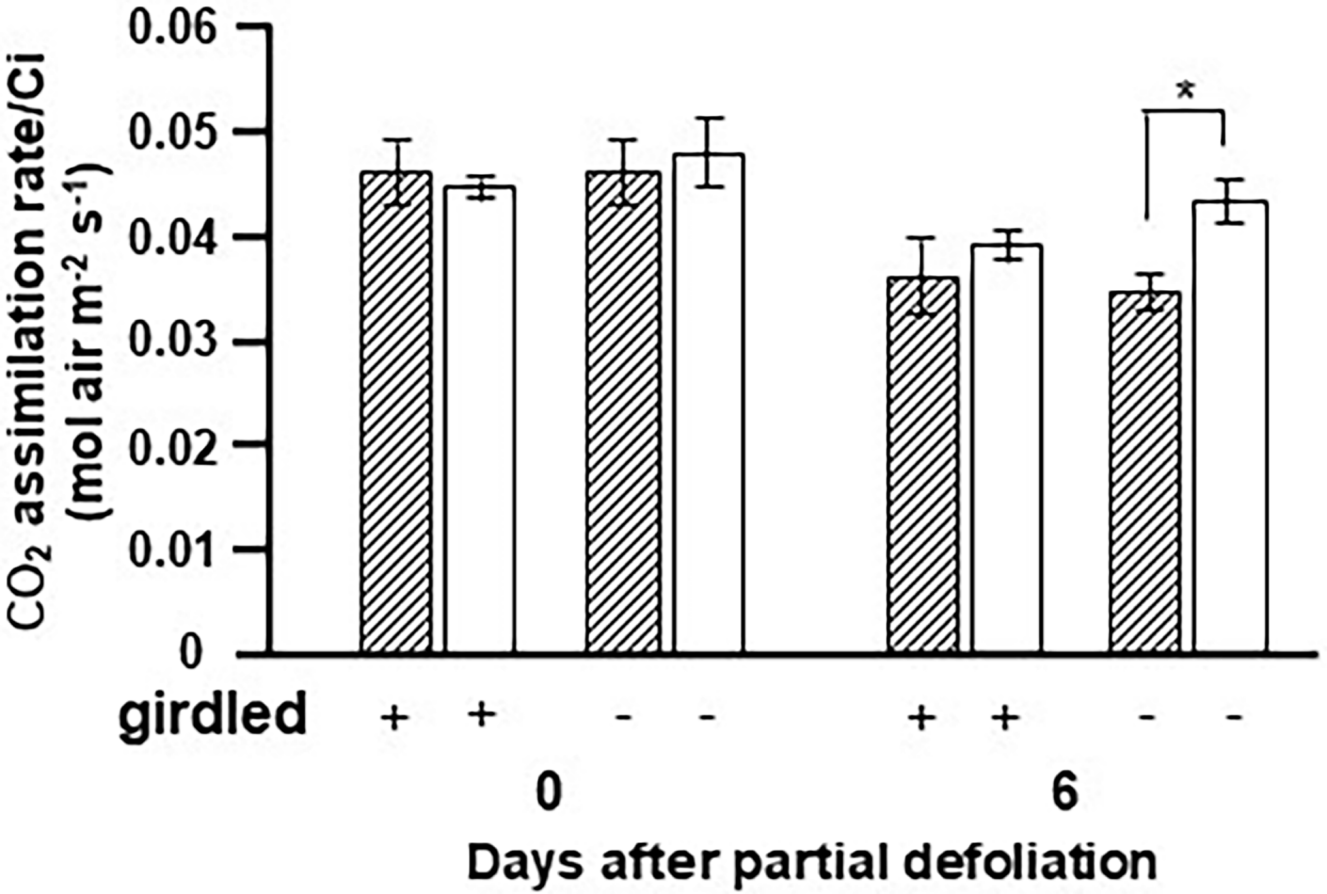
Effect of girdling on the photosynthetic activity response to partial defoliation. Photosynthetic rate per internal CO_2_ concentration (Ci) of source leaves in nondefoliated plants (hatched bars) and the remaining tomato leaves after partial defoliation of the other leaves (empty bars). Defoliation was performed on mature variety M82 plants at the reproductive stage. Lower stem girdling was performed immediately after partial defoliation. Plants were grown in a temperature-controlled greenhouse (25°C/18°C day/night). Data represent means (±SE) of five biological replicates. Asterisks indicate significant differences between partially defoliated and nondefoliated plants at *p* < 0.05 by Student’s *t*-test.

The fact that upregulation of photosynthesis following partial defoliation is associated with altered CK biosynthesis in the roots (Glanz-Idan et al., 2020), and our current findings that overexpression of *IPT* in the roots affects source leaf functioning, suggest that this response is associated with long-distance shoot–root signaling. Plant girdling disrupts this long-distance communication (Supplementary Figure S1). The next set of experiments was aimed at exploring the effect of girdling on the photosynthetic response to partial defoliation. Due to the effect of girdling on transpiration and stomatal conductance, we measured the carboxylation efficiency using the ratio of photosynthesis to Ci as a parameter. As expected, partial defoliation resulted in upregulation of this ratio by over 25% after 6 days (Figure 5). However, the effect of partial defoliation was eliminated by girdling. Similar values were obtained in source leaves of control nongirdled (nondefoliated) and both types of girdled plants: defoliated and nondefoliated. These results suggest that phloem (and possibly xylem) integrity is required to maintain a continuous flow of information between source leaves and roots, in order to induce upregulation of photosynthesis in response to alterations in source–sink relationships.

## Discussion

We previously established that leaf senescence is delayed when the source-to-sink ratio is reduced by partial defoliation (Glanz-Idan et al., 2020). We proposed that this response is associated with elicitation of a root–shoot signaling network and alteration of CK biosynthesis. Our current findings indicate that leaf senescence is delayed *via* manipulation of CK biosynthesis specifically in the roots.

The role of CKs in regulating growth and developmental processes has been well documented (for reviews see Mok and Mok, 2001; Miyawaki et al., 2004; Zurcher and Müller, 2016). Over 60 years ago, Richmond and Lang (1957) demonstrated the CKs’ effect in alleviating leaf senescence, and more recent studies have established their involvement in the regulation of photosynthetic activity, including development and structural differentiation of chloroplasts and inhibition of chlorophyll degradation (Cortleven and Schmulling, 2015). Overexpression of *IPT,* under both the *MDK* and *FBPase* promoters, raised total CK levels in source leaves (Figure 3, Supplementary Figure S5). Nevertheless, delayed leaf senescence, measured as the maintenance of photosynthetic activity in aging source leaves, was only observed in MDK:IPT plants (Table 2; Figure 1). This result was somewhat surprising considering that the total CK level in leaves of MDK:IPT plants was significantly lower than that measured in leaves of FBPase:IPT plants (Figure 3). Contradictory effects on chlorophyll content and fluorescence have been observed with the application of different aromatic CKs to apple leaves (Dobranszki and Mendler-Drienyovszki, 2014). Overexpression of *IPT* under a senescence-associated *Arabidopsis* gene (*SAG12*) promoter inhibited senescence of old tobacco leaves (Gan and Amasino, 1995, 1996). A similar effect on chlorophyll content and photosynthesis was demonstrated in old tomato leaves expressing *IPT* under the *SAG12* promoter (Swartzberg et al., 2006). In both cases, expression of the heterologous *IPT* was detected only in the old leaves. This promoter enabled spatial and temporal control over expression of the target genes at the last stage of leaf development. Gan and Amasino (1995) proposed that delayed senescence is controlled by autoregulation of CK concentration. Inhibition of leaf senescence reduced the influence of the SAG promoter and the expression of IPT such that CK concentration was increased to the minimal level required to delay senescence. These results support our findings and indicate that CK-imposed delayed senescence is not correlated with the level of total CKs accumulated in the leaf; molecular control over leaf aging is more complicated than that.

Similar to our previous findings, partial defoliation resulted in upregulation of photosynthesis (Figure 1; Glanz-Idan et al., 2020). Overexpression of *IPT* in either source leaves or roots eliminated the influence of partial defoliation (Figure 1). These results indicate the involvement of CK biosynthesis in the photosynthetic response to altered source–sink relationships. More detailed analyses of CKs in the source leaves revealed that the concentrations of all three major CK types were highest in FBPase:IPT plants; however, this was mainly due to massive accumulation of iPR (Supplementary Figure S5). Interestingly, leaf concentrations of *t*ZR and isopentenyladenosine-5′-monophosphate (iPMP) were highest in MDK:IPT plants (Supplementary Figure S5), indicating that overexpression of *IPT* under a root-specific promoter has a specific and different influence on CKs metabolism in source leaves compared to its overexpression under a source leaf-specific promoter. It is possible that overexpression of *IPT* in the root triggers an alteration in the biosynthetic pathway of CKs in the leaves such that the conversion to iPR is reduced, resulting in higher accumulation of iPMP.

The molecular mechanism by which CKs control leaf aging has yet to be explored. We propose that root-to-shoot CK-derived signaling is activated due to *IPT* overexpression in the roots, resulting in the specific spatial and quantitative alteration in leaf CK biosynthesis that is required to delay senescence.

One main question concerns the nature of this root-to-shoot signal. An obvious candidate is a CK derivative. As *t*Z and *t*ZR are the main CKs translocating in the xylem from root to shoot (Osugi et al., 2017), it is logical to consider them as primary candidates. Interestingly, the concentration of total CKs in the roots of MDK:IPT and FBPase:IPT plants was similar (Figure 3), but the concentrations of iP9G, *t*Z9G, and *c*ZR were significantly higher when *IPT* was overexpressed under the root-specific promoter (Supplementary Figure S6). *c*Z derivatives are more abundant in the phloem (Corbesier et al., 2003; Hirose et al., 2008), where the flow is from shoot to root, but *c*ZR also makes up about 2% of the xylem sap CKs (Osugi et al., 2017), making it a potential signaling agent. The biological role of glucose-conjugated CKs such as iP9G and *t*Z9G is still not known and in general, they are considered to be inactive. Hallmark et al. (2020) recently provided experimental evidence for the ability of *t*Z9G to delay senescence of *Arabidopsis* cotyledons. This new finding may point to a potential role for *t*Z9G as a root-borne signal involved in the control of leaf aging. Nevertheless, it is quite possible that the root-to-shoot signal is not a CK derivative but an alternative molecule whose expression in the root is stimulated by overexpression of *IPT* and alteration of CK biosynthesis.

While expression of *IPT* under the *AtMDK* promoter had a major effect on leaf senescence, the reduction in photosynthetic activity with leaf aging was similar for MDK:CKX, FBPase:CKX and control plants (Figure 2). Despite the tissue-specific overexpression (Supplementary Figure S7), CK concentrations in roots and leaves of our FBPase:CKX and MDK:CKX plants were similar to those measured in control plants, which could explain the similar photosynthetic activities (Supplementary Tables S1, S2). These results may relate to autoregulation of CK concentration such that CKs degradation activates higher expression/activity of CK-metabolizing enzymes. The gene selected for our study was *AtCKX3*. Overexpression of this gene under the CaMV-35S resulted in longer roots and inhibited shoots growth of transgenic Arabidopsis plants (Werner et al., 2003) Nevertheless, we cannot rule out the possibility that the selected gene does not have similar influence in tomato plants and an alternative *CKX* gene would impose more significant effect on CK degradation.

**Figure 6:**
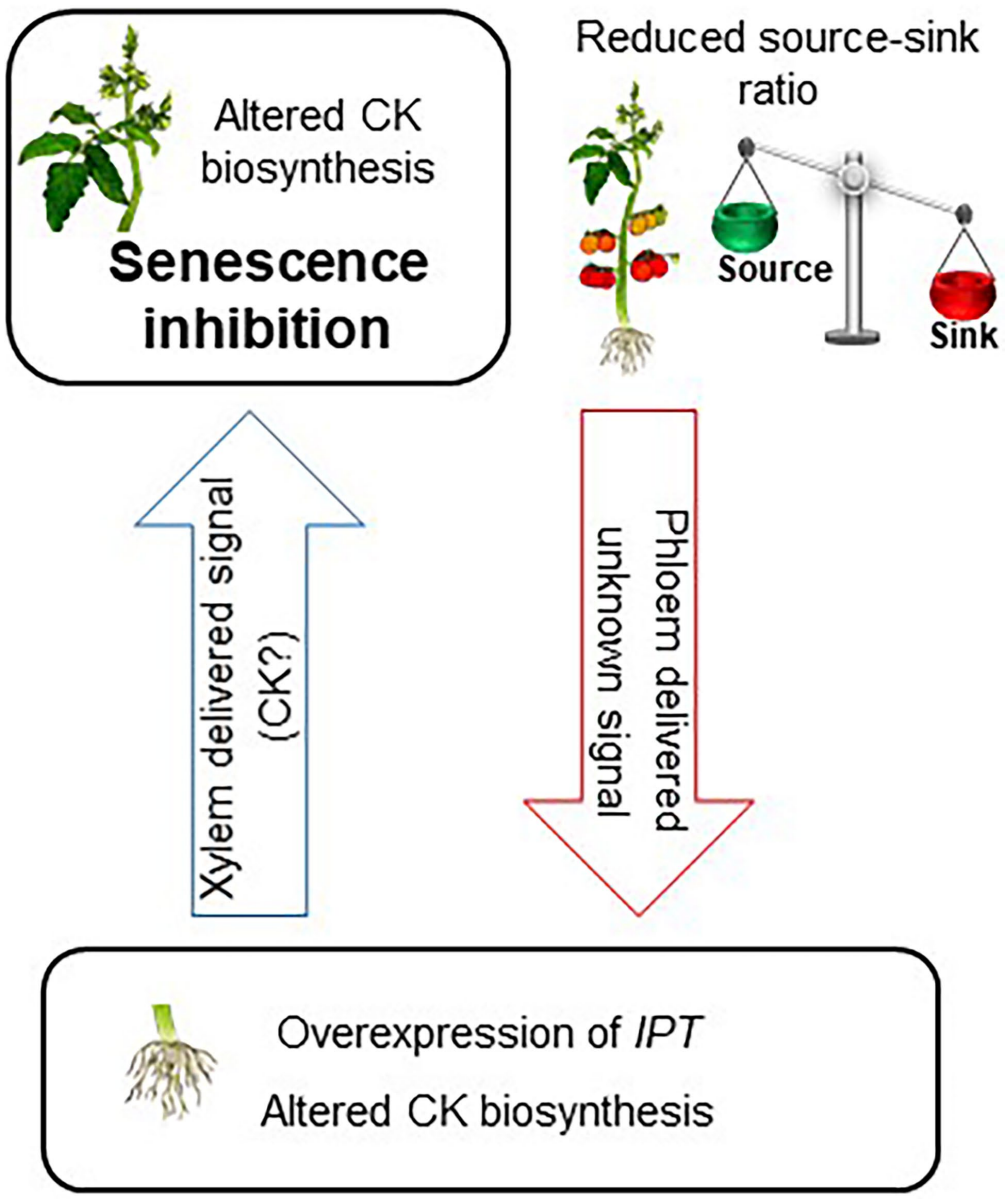
Model for root CK biosynthesis effect on the functioning and aging of source leaves. Reducing source–sink ratio or overexpression of IPT under a root-specific promoter leads to altered CK biosynthesis in the roots. This change in root CK metabolism elicits root-to-shoot translocation of a signaling agent to promote alteration in CK biosynthesis in the leaves. Specific spatial and quantitative accumulation of CK results in delayed senescence of source leaves.

The presented results demonstrate the effect of root CK biosynthesis on the functioning and aging of source leaves. We suggest that production of additional CKs specifically in the roots, by overexpression of *IPT* or by partial defoliation, elicits root-to-shoot translocation of a signaling agent to promote alterations in CK biosynthesis in the source leaves, resulting in upregulation of photosynthesis and delayed leaf senescence. As the effect of partial defoliation is eliminated by stem girdling, we propose that roots sense partial defoliation by a signal translocating from shoot to root *via* the phloem (Figure 6). Further research should aim at identifying and characterizing the nature of the various signaling molecules and their molecular mechanism of long-distance transport.

## Data Availability Statement

The original contributions presented in the study are included in the article/Supplementary Material, further inquiries can be directed to the corresponding author.

## Author Contributions

NG-I and SW conceived and designed the study. NG-I, ML, PT, and OV designed and conducted the experiments. NG-I, ML, and PT analyzed the data. NG-I and SW wrote the manuscript. All authors contributed to the article and approved the submitted version.

## Funding

This research was partially supported by the Israel Ministry of Agriculture and Rural Development (Eugene Kandel Knowledge Centers) as part of the Root of the Matter—The root zone knowledge center for leveraging modern agriculture. This paper is a contribution from the Uri Kinamon Laboratory. NG-I was supported by a scholarship from the Uri Kinamon Foundation. PT and OV were supported by ERDF project “Plants as a tool for sustainable global development” (CZ.02.1.01./0.0/0.0/16_019/0000827) and project no. RO0418 funded by Ministry of Agriculture, Czechia.

## Conflict of Interest

The authors declare that the research was conducted in the absence of any commercial or financial relationships that could be construed as a potential conflict of interest.

## Publisher’s Note

All claims expressed in this article are solely those of the authors and do not necessarily represent those of their affiliated organizations, or those of the publisher, the editors and the reviewers. Any product that may be evaluated in this article, or claim that may be made by its manufacturer, is not guaranteed or endorsed by the publisher.
