# Supplementary material for: Delayed Leaf Senescence by Upregulation of Cytokinin Biosynthesis Specifically in Tomato Roots
Source: Front Plant Sci. 2022 Jul 6;13:922106. doi: 10.3389/fpls.2022.922106 (PMC9298850; doi:10.3389/fpls.2022.922106)
Supplement: Supplementary file 1 [file Table_1.DOCX]

**SUPPLEMENTARY TABLES**

**Table S1** Evaluation of cytokinin concentration in source leaves of control and transgenic tomato plants expressing *CKX* under either source leaf-specific (*FBPase*) or root cap-specific (*AtMDK*) promoter. Data represent means (±SE) of six replicates

| **MDK:CKX** | **FBPase:CKX** | **M82** | **Cytokinin concentration in source leaves**  **(pmol g FW^-1^)** |
| --- | --- | --- | --- |
| 0.10 ± 0.03 a | 0.11 ± 0.03 a | 0.20 ± 0.04 a | iP |
| 1.64 ± 0.45 a | 1.75 ± 0.16 a | 2.68 ± 1.03 a | iPR |
| 0.028 ± 0.004 b | 0.098 ± 0.012 a | 0.036 ± 0.003 b | iP9G |
| n.d. | n.d. | n.d. | iPMP |
| **1.77 ± 0.47 a** | **1.96 ± 0.17 a** | **2.91 ± 1.01 a** | **Total iP type** |
| 1.87 ± 0.45 a | 0.87 ± 0.34 a | 0.74 ± 0.36 a | *t*Z |
| 0.41 ± 0.07 a | 0.47 ± 0.09 a | 0.35 ± 0.03 a | *t*ZR |
| n.d. | n.d. | n.d. | *t*Z9G |
| **2.29 ± 0.43 a** | **1.34 ± 0.32 a** | **1.09 ± 0.35 a** | **Total *t*Z type** |
| 0.66 ± 0.20 a | 0.61 ± 0.10 a | 0.79 ± 0.21 a | *c*Z |
| 5.70 ± 2.02 a | 7.29 ± 1.21 a | 5.82 ± 1.89 a | *c*ZR |
| n.d. | n.d. | n.d. | *c*Z9G |
| **6.36 ± 2.02 a** | **7.90 ± 1.12 a** | **6.61 ± 1.86 a** | **Total *c*Z type** |
| **10.41 ± 2.29 a** | **11.19 ± 1.24 a** | **10.62 ± 2.79 a** | **Total cytokinins** |

Different letters indicate significant differences between control and the respective transgenic plants by Student’s t test, *P* < 0.05.

**Table S2** Evaluation of cytokinin concentration in roots of control and transgenic tomato plants expressing *CKX* under either source leaf-specific (*FBPase*) or root cap-specific (*AtMDK*) promoter. Data represent means (±SE) of six replicates

| **MDK:CKX** | **FBPase:CKX** | **M82** | **Cytokinin concentration in roots**  **(pmol g FW^-1^)** |
| --- | --- | --- | --- |
| 0.05 ± 0.02 a | 0.06 ± 0.01 a | 0.09 ± 0.01 a | iP |
| 1.13 ± 0.33 a | 1.16 ± 0.25 a | 1.63 ± 0.29 a | iPR |
| 0.21 ± 0.04 a | 0.24 ± 0.02 a | 0.28 ± 0.05 a | iP9G |
| n.d. | n.d. | n.d. | iPMP |
| **1.38 ± 0.38 a** | **1.47 ± 0.25 a** | **1.99 ± 0.35 a** | **Total iP type** |
| 0.17 ± 0.05 b | 0.73 ± 0.20 a | 0.16 ± 0.02 b | *t*Z |
| 2.63 ± 1.03 a | 2.38 ± 0.49 a | 2.59 ± 0.71 a | *t*ZR |
| 0.53 ± 0.11 a | 0.74 ± 0.08 a | 0.72 ± 0.17 a | *t*Z9G |
| **3.32 ± 1.10 a** | **3.85 ± 0.58 a** | **3.47 ± 0.88 a** | **Total *t*Z type** |
| 0.24 ± 0.07 a | 0.19 ± 0.08 a | 0.18 ± 0.02 a | *c*Z |
| 1.08 ± 0.39 a | 0.45 ± 0.06 a | 0.70 ± 0.15 a | *c*ZR |
| n.d. | n.d. | n.d. | *c*Z9G |
| **1.32 ± 0.42 a** | **0.64 ± 0.14 a** | **0.87 ± 0.16 a** | **Total *c*Z type** |
| **6.02 ± 1.25 a** | **5.95 ± 0.79 a** | **6.33 ± 1.27 a** | **Total cytokinins** |

Different letters indicate significant differences between control and the respective transgenic plants by Student’s t test, *P* < 0.05.
