# Supplementary material for: Delayed Leaf Senescence by Upregulation of Cytokinin Biosynthesis Specifically in Tomato Roots
Source: Front Plant Sci. 2022 Jul 6;13:922106. doi: 10.3389/fpls.2022.922106 (PMC9298850; doi:10.3389/fpls.2022.922106)
Supplement: Supplementary file 2 [file Presentation_1.PPTX]

## Slide 1
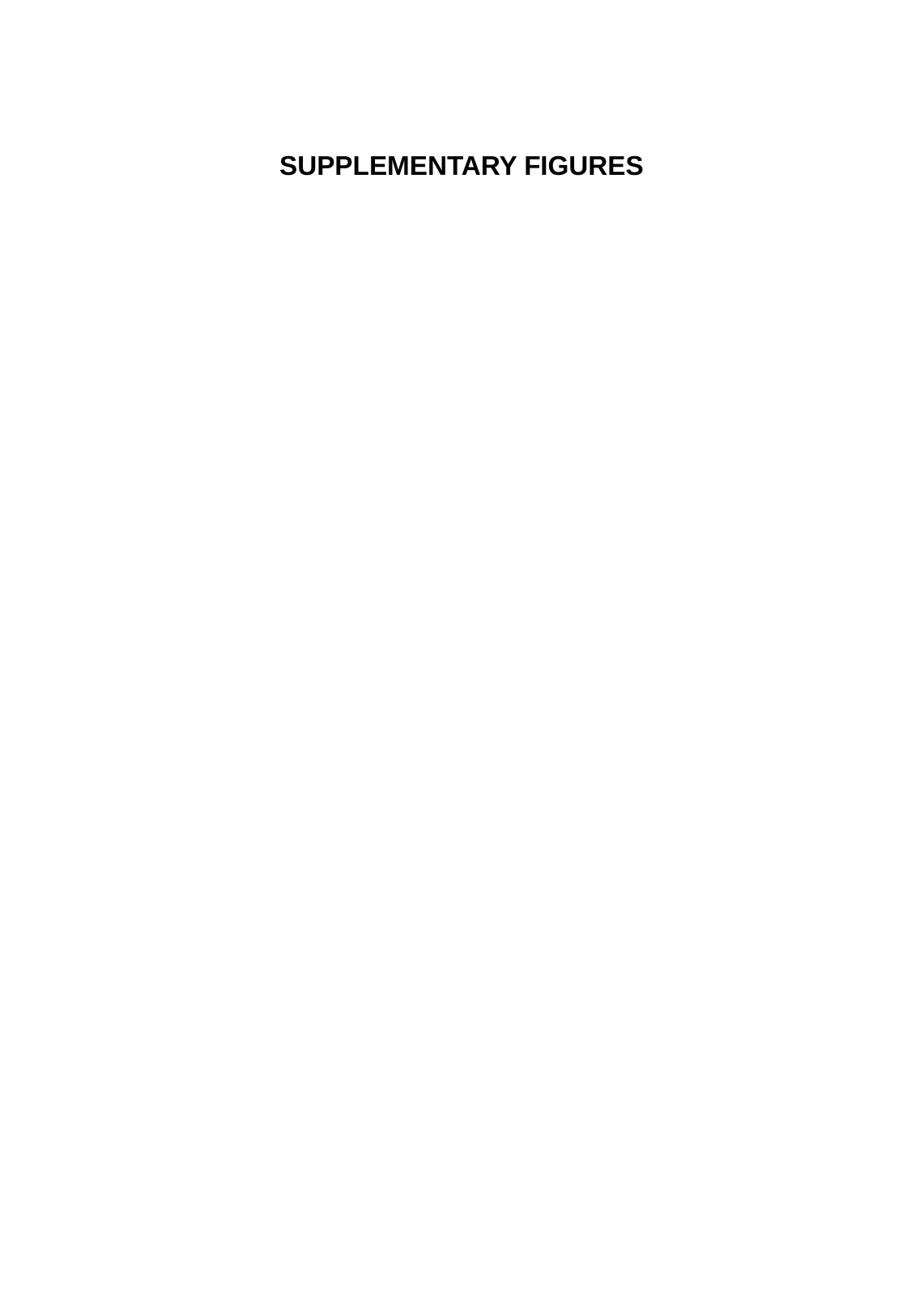

SUPPLEMENTARY FIGURES

## Slide 2
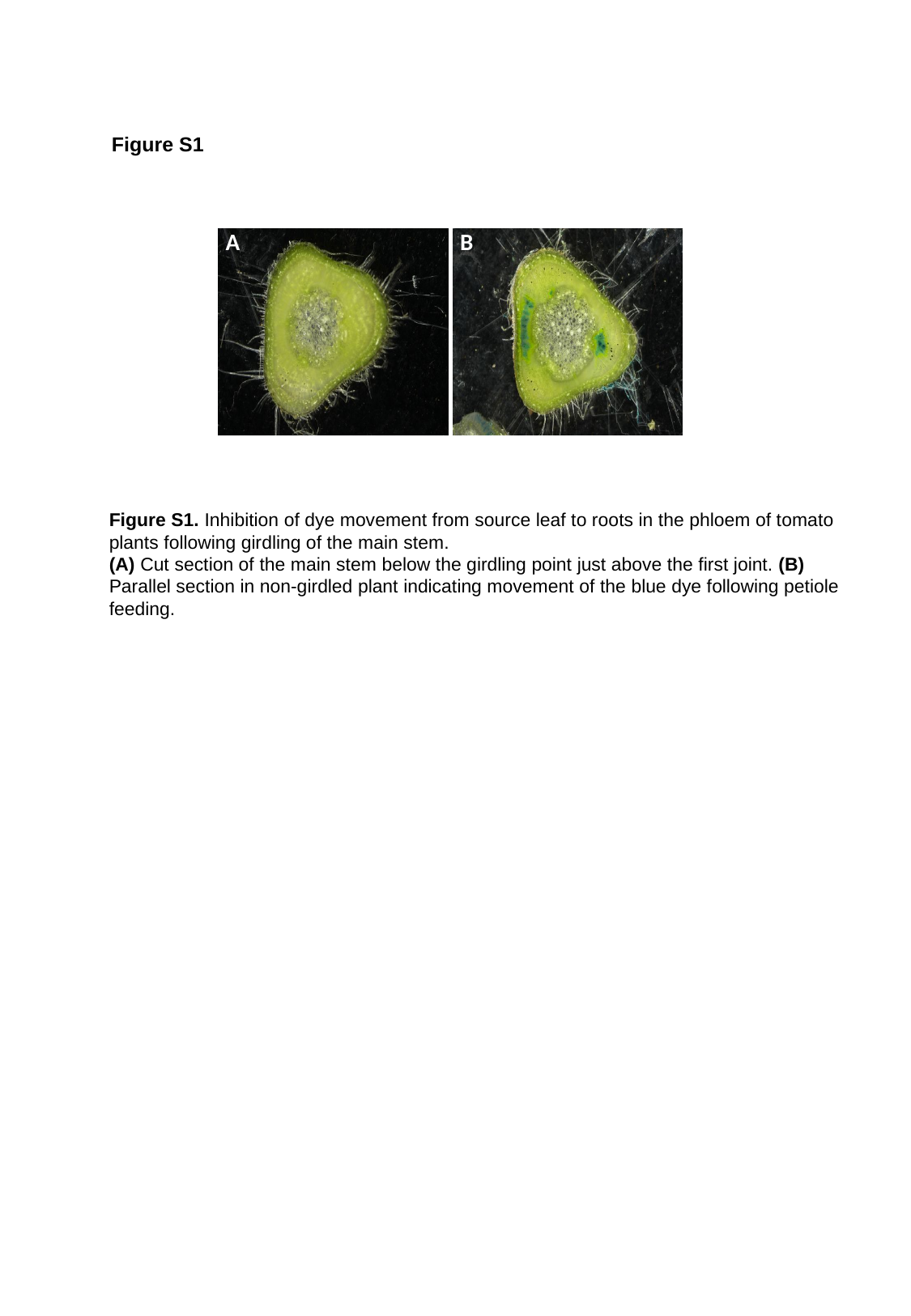

Figure S1
A
B
Figure S1. Inhibition of dye movement from source leaf to roots in the phloem of tomato plants following girdling of the main stem.
(A) Cut section of the main stem below the girdling point just above the first joint. (B) Parallel section in non-girdled plant indicating movement of the blue dye following petiole feeding.

## Slide 3
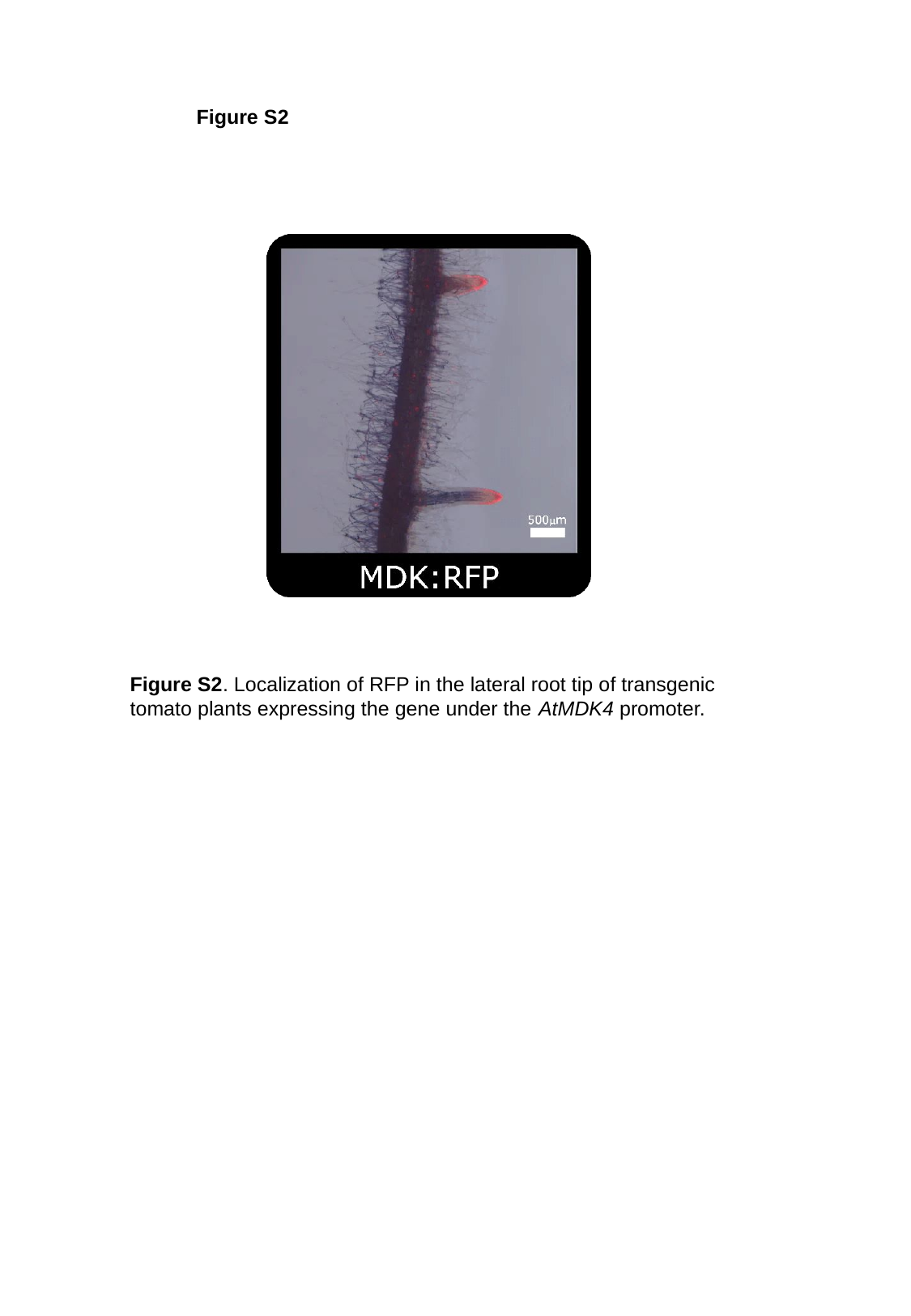

Figure S2
Figure S2. Localization of RFP in the lateral root tip of transgenic tomato plants expressing the gene under the AtMDK4 promoter.

## Slide 4
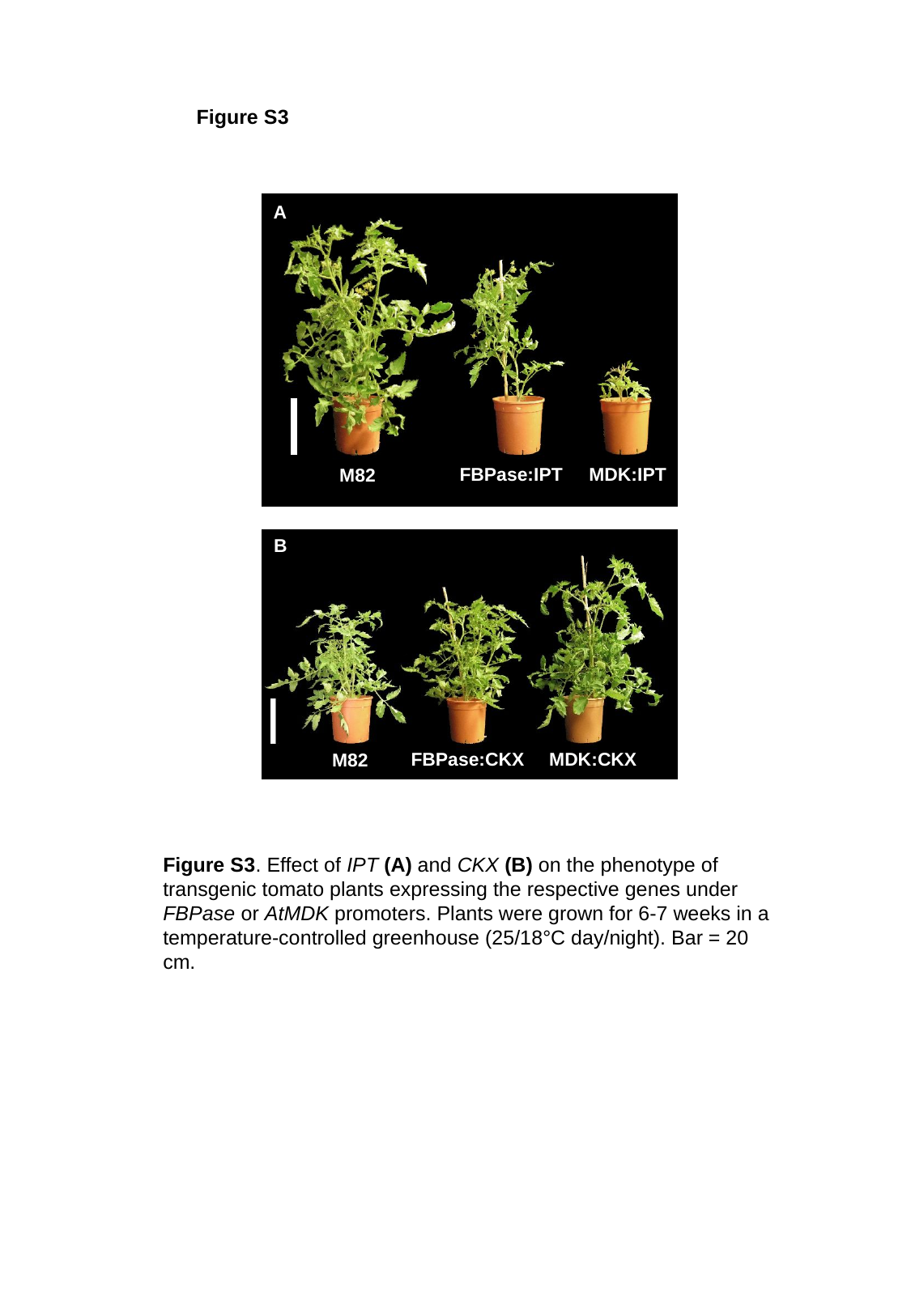

Figure S3
A
FBPase:IPT
MDK:IPT
M82
B
FBPase:CKX
MDK:CKX
M82
Figure S3. Effect of IPT (A) and CKX (B) on the phenotype of transgenic tomato plants expressing the respective genes under FBPase or AtMDK promoters. Plants were grown for 6-7 weeks in a temperature-controlled greenhouse (25/18°C day/night). Bar = 20 cm.

## Slide 5
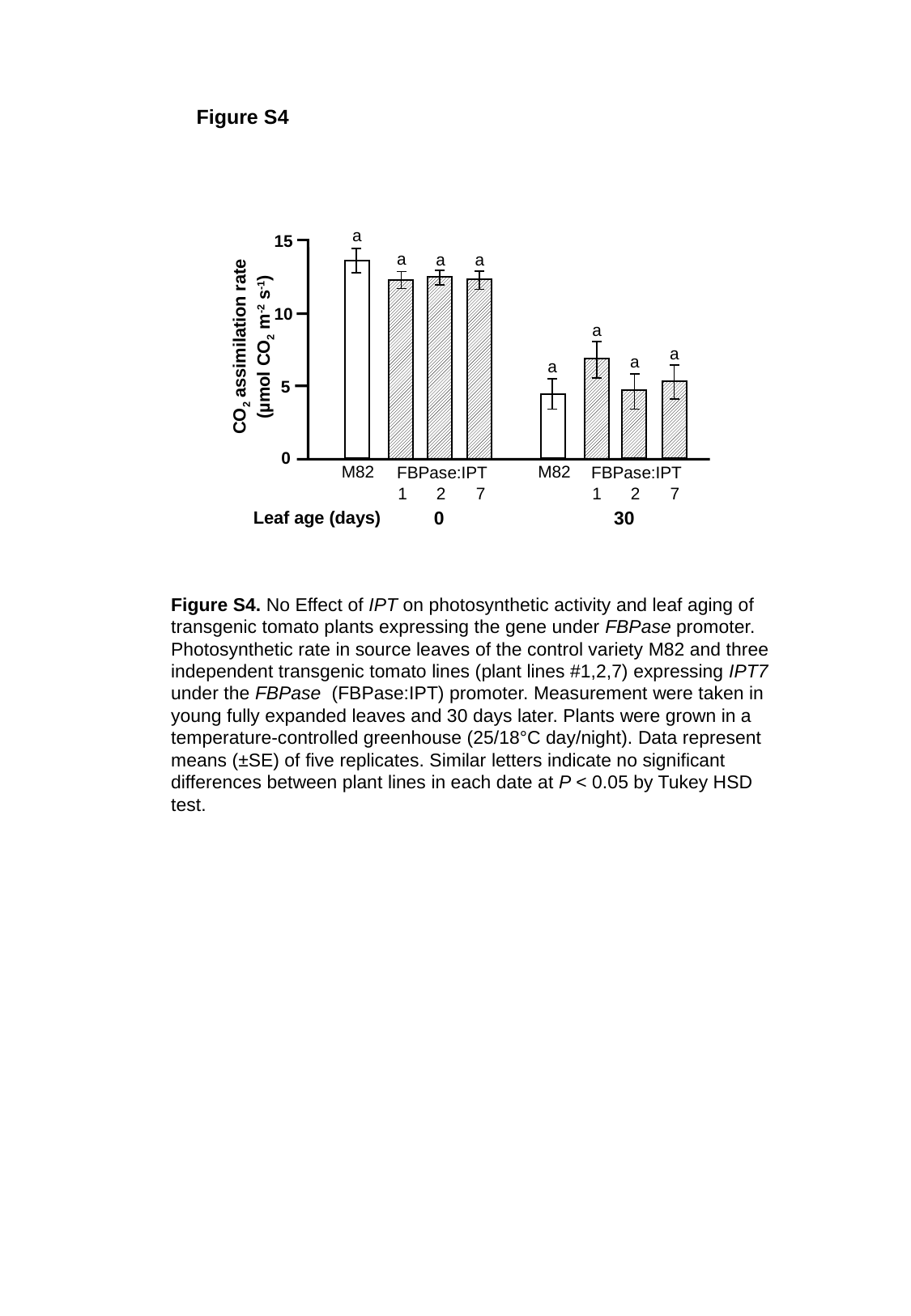

Figure S4
a
15
10
CO2 assimilation rate (µmol CO2 m-2 s-1)
 5
 0
M82
M82
FBPase:IPT
 1
 2
 7
FBPase:IPT
 1
 2
 7
0
 30
Leaf age (days)
a
a
a
a
a
a
a
Figure S4. No Effect of IPT on photosynthetic activity and leaf aging of transgenic tomato plants expressing the gene under FBPase promoter.
Photosynthetic rate in source leaves of the control variety M82 and three independent transgenic tomato lines (plant lines #1,2,7) expressing IPT7 under the FBPase (FBPase:IPT) promoter. Measurement were taken in young fully expanded leaves and 30 days later. Plants were grown in a temperature-controlled greenhouse (25/18°C day/night). Data represent means (±SE) of five replicates. Similar letters indicate no significant differences between plant lines in each date at P < 0.05 by Tukey HSD test.

## Slide 6
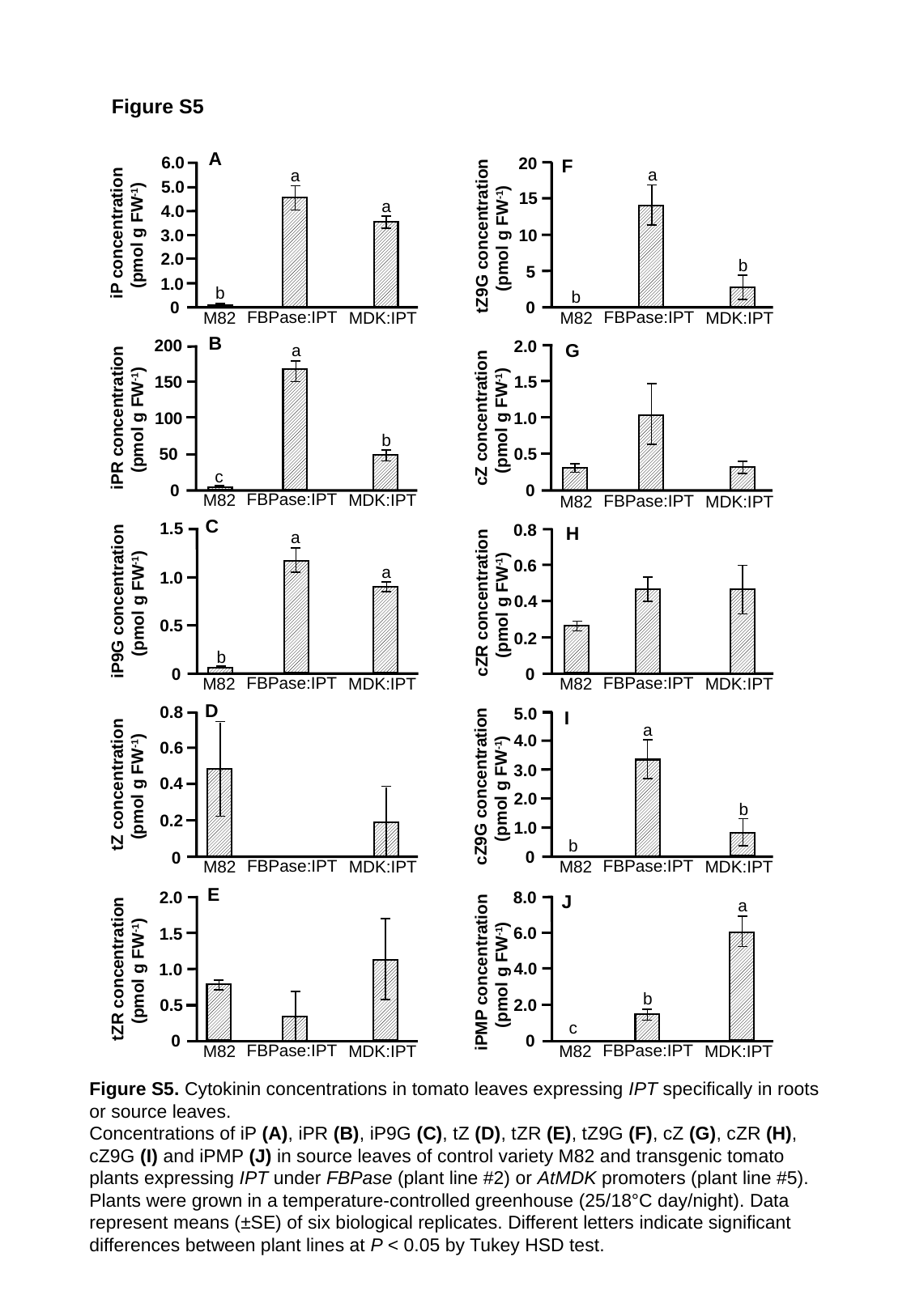

Figure S5
A
6.0
20
F
a
a
5.0
15
a
4.0
iP concentration
(pmol g FW-1)
tZ9G concentration
(pmol g FW-1)
10
3.0
2.0
b
 5
1.0
b
b
 0
0
FBPase:IPT
M82
MDK:IPT
FBPase:IPT
M82
MDK:IPT
B
200
2.0
G
a
1.5
150
iPR concentration
(pmol g FW-1)
cZ concentration
(pmol g FW-1)
100
1.0
b
0.5
50
c
 0
 0
FBPase:IPT
M82
MDK:IPT
FBPase:IPT
M82
MDK:IPT
C
1.5
0.8
H
a
0.6
a
1.0
iP9G concentration
(pmol g FW-1)
cZR concentration
(pmol g FW-1)
0.4
0.5
0.2
b
 0
 0
FBPase:IPT
M82
MDK:IPT
FBPase:IPT
M82
MDK:IPT
D
0.8
5.0
I
a
4.0
0.6
3.0
tZ concentration
(pmol g FW-1)
cZ9G concentration
(pmol g FW-1)
0.4
2.0
b
0.2
1.0
b
 0
 0
FBPase:IPT
M82
MDK:IPT
FBPase:IPT
M82
MDK:IPT
E
2.0
8.0
J
a
6.0
1.5
tZR concentration
(pmol g FW-1)
iPMP concentration
(pmol g FW-1)
4.0
1.0
b
2.0
0.5
c
 0
 0
FBPase:IPT
M82
MDK:IPT
FBPase:IPT
M82
MDK:IPT
Figure S5. Cytokinin concentrations in tomato leaves expressing IPT specifically in roots or source leaves.
Concentrations of iP (A), iPR (B), iP9G (C), tZ (D), tZR (E), tZ9G (F), cZ (G), cZR (H), cZ9G (I) and iPMP (J) in source leaves of control variety M82 and transgenic tomato plants expressing IPT under FBPase (plant line #2) or AtMDK promoters (plant line #5). Plants were grown in a temperature-controlled greenhouse (25/18°C day/night). Data represent means (±SE) of six biological replicates. Different letters indicate significant differences between plant lines at P < 0.05 by Tukey HSD test.

## Slide 7
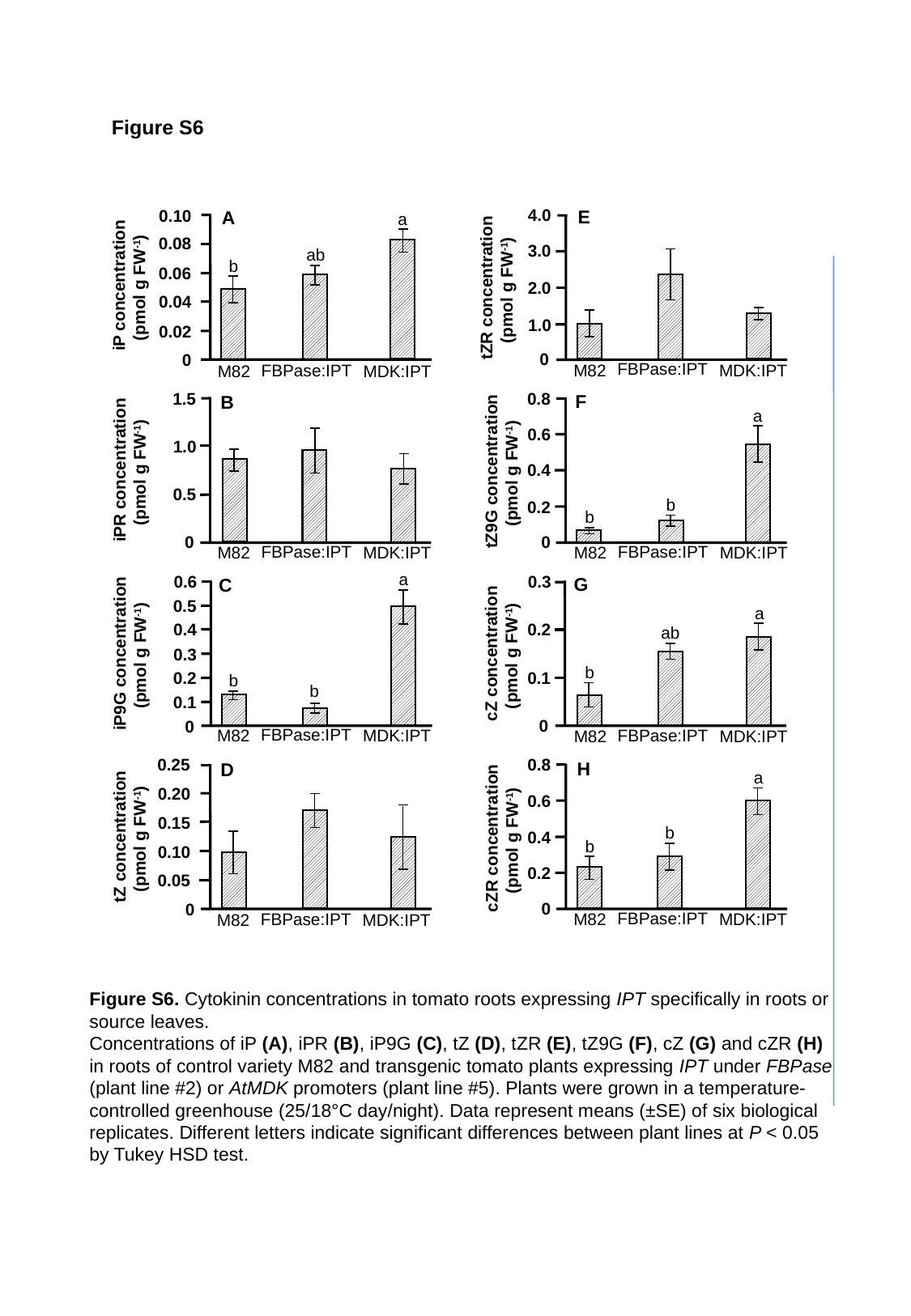

Figure S6
4.0
0.10
E
A
a
0.08
3.0
ab
b
0.06
iP concentration
(pmol g FW-1)
tZR concentration
(pmol g FW-1)
2.0
0.04
1.0
0.02
 0
 0
FBPase:IPT
M82
MDK:IPT
FBPase:IPT
M82
MDK:IPT
1.5
0.8
F
B
a
0.6
1.0
iPR concentration
(pmol g FW-1)
tZ9G concentration
(pmol g FW-1)
0.4
0.5
b
0.2
b
 0
 0
FBPase:IPT
M82
MDK:IPT
FBPase:IPT
M82
MDK:IPT
a
0.3
0.6
G
C
0.5
a
0.2
0.4
ab
iP9G concentration
(pmol g FW-1)
cZ concentration
(pmol g FW-1)
0.3
b
0.1
0.2
b
b
0.1
 0
 0
FBPase:IPT
M82
MDK:IPT
FBPase:IPT
M82
MDK:IPT
0.25
0.8
H
D
a
0.20
0.6
0.15
tZ concentration
(pmol g FW-1)
cZR concentration
(pmol g FW-1)
b
0.4
b
0.10
0.2
0.05
 0
 0
FBPase:IPT
M82
MDK:IPT
FBPase:IPT
M82
MDK:IPT
Figure S6. Cytokinin concentrations in tomato roots expressing IPT specifically in roots or source leaves.
Concentrations of iP (A), iPR (B), iP9G (C), tZ (D), tZR (E), tZ9G (F), cZ (G) and cZR (H) in roots of control variety M82 and transgenic tomato plants expressing IPT under FBPase (plant line #2) or AtMDK promoters (plant line #5). Plants were grown in a temperature-controlled greenhouse (25/18°C day/night). Data represent means (±SE) of six biological replicates. Different letters indicate significant differences between plant lines at P < 0.05 by Tukey HSD test.

## Slide 8
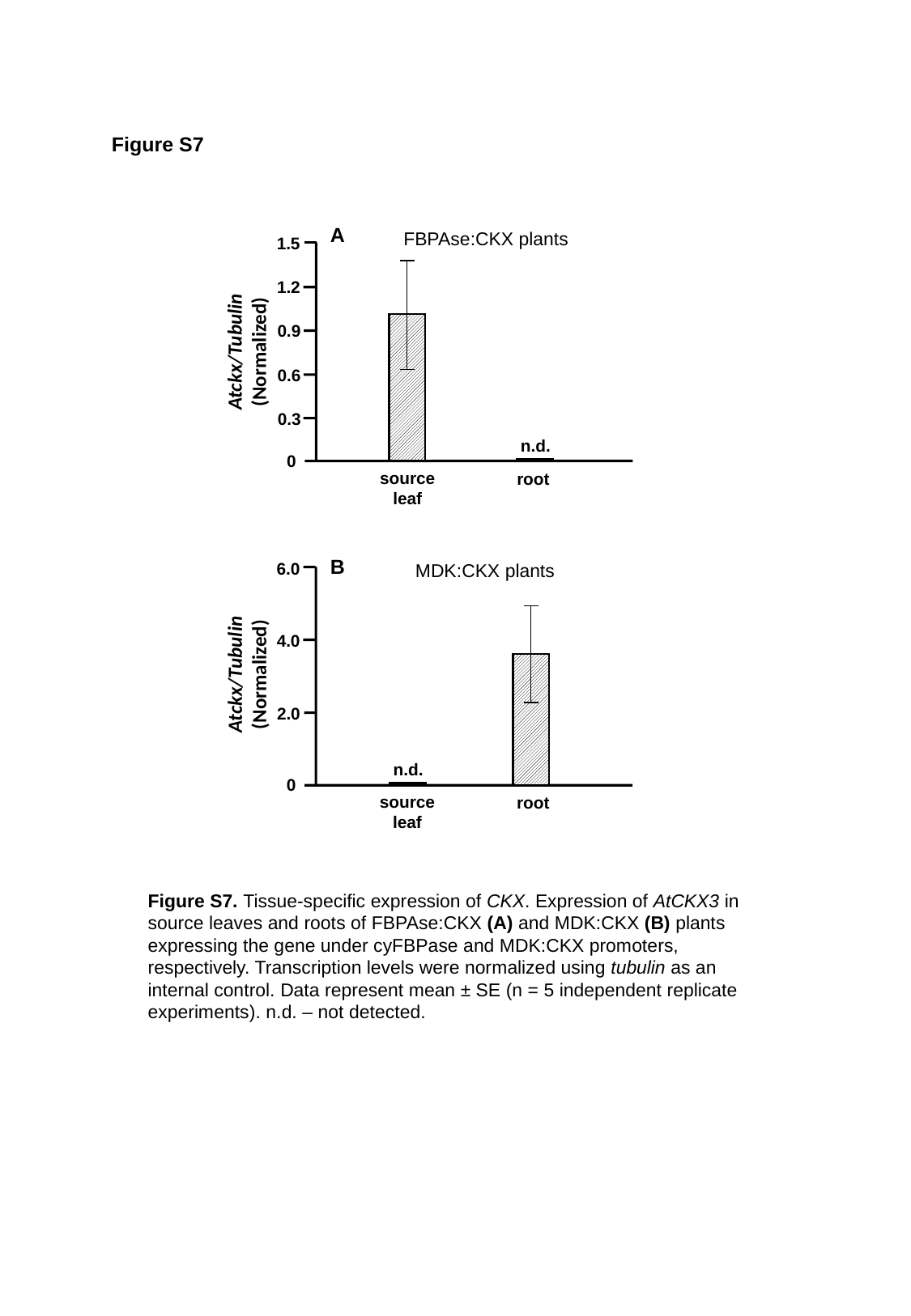

Figure S7
A
FBPAse:CKX plants
1.5
1.2
0.9
Atckx/Tubulin (Normalized)
0.6
0.3
n.d.
 0
source leaf
root
B
6.0
MDK:CKX plants
4.0
Atckx/Tubulin (Normalized)
2.0
n.d.
 0
source leaf
root
Figure S7. Tissue-specific expression of CKX. Expression of AtCKX3 in source leaves and roots of FBPAse:CKX (A) and MDK:CKX (B) plants expressing the gene under cyFBPase and MDK:CKX promoters, respectively. Transcription levels were normalized using tubulin as an internal control. Data represent mean ± SE (n = 5 independent replicate experiments). n.d. – not detected.

## Slide 9
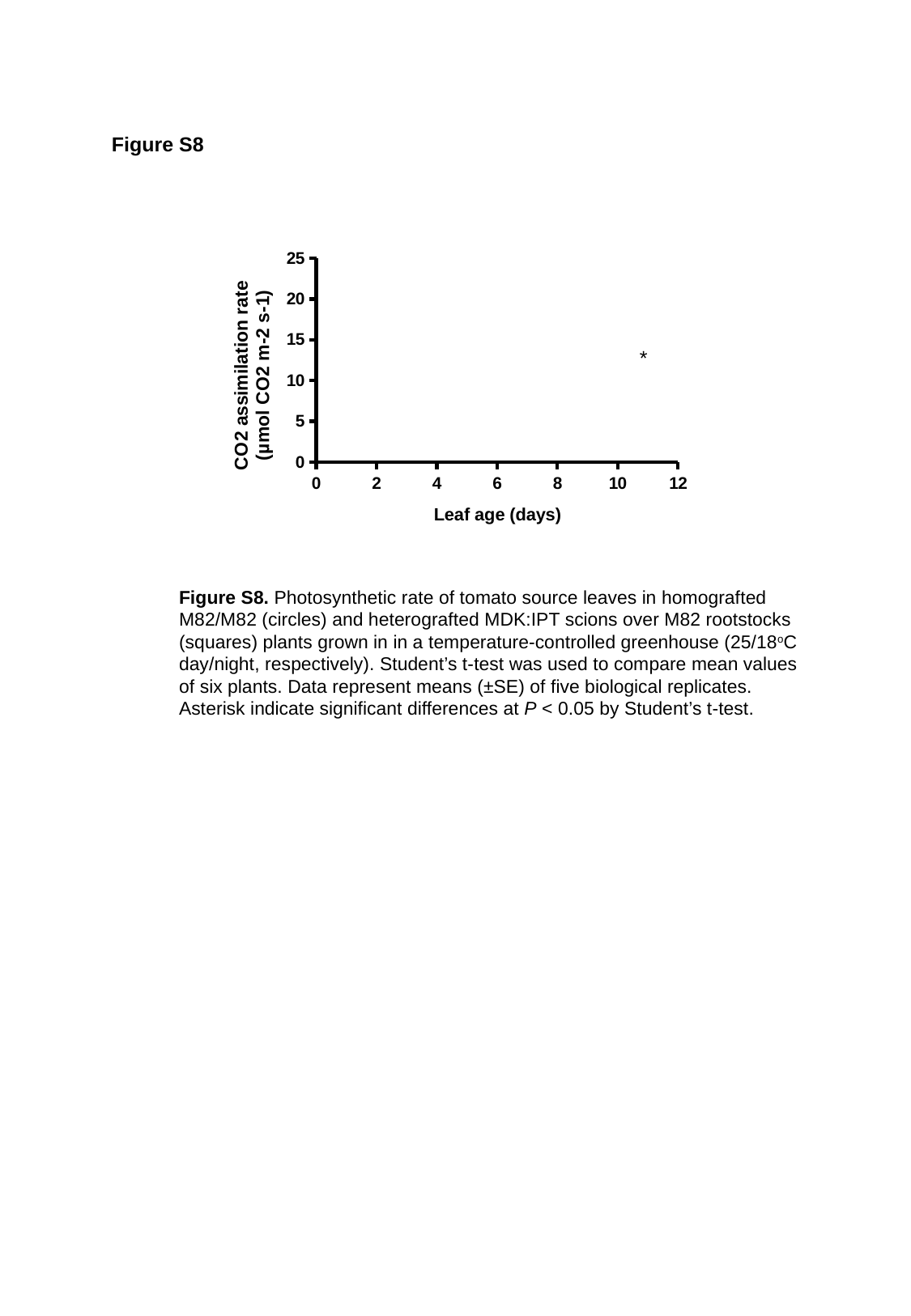

Figure S8
### Chart
| Category | M82/M82 | MDK-IPT/M82 |
|---|---|---|Leaf age (days)
*
Figure S8. Photosynthetic rate of tomato source leaves in homografted M82/M82 (circles) and heterografted MDK:IPT scions over M82 rootstocks (squares) plants grown in in a temperature-controlled greenhouse (25/18oC day/night, respectively). Student’s t-test was used to compare mean values of six plants. Data represent means (±SE) of five biological replicates. Asterisk indicate significant differences at P < 0.05 by Student’s t-test.
